# Antioxidant and Anti-Inflammatory Activities of High-Glucosinolate-Synthesis Lines of *Brassica rapa*

**DOI:** 10.3390/antiox12091693

**Published:** 2023-08-30

**Authors:** Hyunjin Choi, Hail Kim, Sanghee Han, Hyun Woo Park, In Jin Ha, Jung Sun Kim, Seok-Geun Lee

**Affiliations:** 1 Department of Biomedical Science and Technology, Kyung Hee University, Seoul 02447, Republic of Korea; hjc517@khu.ac.kr; 2 Graduate School, Kyung Hee University, Seoul 02447, Republic of Korea; khi3598@khu.ac.kr (H.K.); sanghee8757@khu.ac.kr (S.H.); ijha@khu.ac.kr (I.J.H.); 3 Genomic Division, Department of Agricultural Bio-Resources, National Institute of Agricultural Sciences, Rural Development Administration, Jeonju 54874, Republic of Korea; hwpark0803@korea.kr

**Keywords:** *Brassica rapa*, glucosinolate, doubled haploid lines, antioxidant, inflammation, NRF2

## Abstract

Excessive oxidative stress and inflammatory responses are associated with the development of various diseases, including cancer. Glucosinolates (GSLs) are phytochemicals known for their antioxidant properties, and doubled haploid lines (DHLs) of *Brassica rapa* with high GSL contents (HGSL) were intentionally developed from two edible subspecies of *Brassica rapa*: *B. rapa* subsp. *trilocularis* and *B. rapa* subsp. *chinensis*. The purpose of the present study is to assess the capacity of HGSL DHLs to mitigate oxidative stress and inflammation in lipopolysaccharide (LPS)-stimulated RAW264.7 cells, compared to pak choi as a parental control. Our findings demonstrate that HGSL DH lines effectively suppressed the expression of inducible nitric oxide synthase, leading to the reduced levels of nitric oxide at non-toxic concentrations. Additionally, these lines exhibited scavenging activity against reactive oxygen species and free radicals. The enhanced antioxidant capacity of HGSL DHLs was mechanistically attributed to the upregulation of antioxidant enzymes, such as NADPH quinone oxidoreductase 1 (NQO1), the glutamate–cysteine ligase catalytic subunit (GCLC), and heme oxygenase-1 (HMOX1). Furthermore, we confirmed that these effects were mediated through the nuclear factor erythroid 2-related factor 2 (NRF2) signaling pathway via p38 phosphorylation. Moreover, HGSL DHLs demonstrated inhibitory effects on pro-inflammatory cytokines and signal transducers and activators of transcription 3 (STAT3) phosphorylation. Collectively, our results indicate that HGSL DHLs possess better antioxidant and anti-inflammatory properties compared to the parental control pak choi in LPS-stimulated RAW264.7 cells, suggesting that HGSL DHLs of *Brassica rapa* could be considered as a beneficial daily vegetable for reducing the risk of inflammation-associated diseases.

## 1. Introduction

Macrophages, as one of the primary immunocytes, play a crucial role in the innate immune response [1]. Their involvement in host defense and inflammatory responses stems from their ability to recognize molecular patterns associated with pathogens through Toll-like receptors (TLRs) [2]. Lipopolysaccharide (LPS) is a component of Gram-negative bacterial cell walls and is often used to induce inflammation in macrophages. LPS acts as a ligand for Toll-like receptor 4 (TLR4), activating downstream signaling pathways [3]. In LPS-stimulated macrophages, inflammation is induced through various pathways including nuclear factor-κB (NF-κB), mitogen-activated protein kinases (MAPK), Janus kinase 2/signal transducers and activators of transcription 3 (JAK2/STAT3), and an increase in reactive oxygen species (ROS) production [4,5]. While inflammation is a necessary defense response to harmful factors such as pathogens and damaged cells, excessive and chronic inflammation can contribute to the development of diverse diseases, including cancer [6,7,8].

LPS not only triggers the inflammatory response but also stimulates the production of ROS [2,3,4,5]. Oxygen metabolism holds a pivotal role in the pathogenesis of inflammation, with ROS being a key molecular mechanism underlying inflammatory-related diseases. The activation of ROS along MAPK and NF-κB fosters inflammation and causes damage to cells and tissues due to their high reactivity [6,7,8,9]. Nuclear factor erythroid 2-related factor 2 (NRF2) acts as a critical regulator in oxidative stress and the inflammatory response, protecting cells against the stress conditions [6,9]. Under homeostatic conditions, NRF2 is inactivated by its negative regulator, Kelch-like ECH-associated protein (KEAP1) [10]. However, under stress conditions, such as LPS stimulation, NRF2 dissociates from KEAP1, translocates to the nucleus, and induces the expression of genes encoding antioxidant enzymes, including heme oxygenase-1 (HMOX1), catalase, NADPH quinone oxidoreductase 1 (NQO1), and the glutamate–cysteine ligase catalytic subunit (GCLC) [11,12].

Epidemiological studies have consistently demonstrated the effectiveness of cruciferous vegetables (Brassicaceae) in preventing various diseases, with these health benefits attributed to a rich and diverse array of secondary metabolites [13]. *Brassica rapa*, a species of the Brassicaceae family, also contains secondary metabolites such as carotenoids, flavonoids, anthocyanins, and glucosinolates (GSLs) [14,15]. GSLs are hydrolyzed by myrosinase, leading to the formation of isothiocyanates (ITCs), such as benzyl isothiocyanate (BITC), phenyl isothiocyanate (PEITC), and sulforaphane (SFN) [14]. GSLs and their breakdown products have been found to possess anticarcinogenic and antioxidative activities [16,17,18]. SFN has been reported to be effective against cardiovascular disease through the activation of NRF2 [19].

In a previous study, many doubled haploid lines (DHLs) were generated from two *Brassica rapa* subspecies that are edible: *B. rapa* subsp. *trilocularis* (yellow sarson) and *B. rapa* subsp. *chinensis* (pak choi) [20]. The DHLs selected for their high GSL contents, between 44.12 and 57.04 µmol/g·dry weight (dw), were designated as high GSL (HGLS)-containing DHLs (HGSL DHLs), with the expectation that they would exhibit improved antioxidant and anti-inflammatory properties compared to the parental *Brassica rapa* [20]. Moreover, in a separate study, three edible HGSL DHLs (DH005, DH014, and DH026) demonstrated enhanced anticancer effects in human colorectal cancer cells compared to pak choi [21]. Given the well-established association between colorectal cancer and inflammation, as well as the known beneficial properties of GSLs, it is reasonable to hypothesize that these HGLS DHLs may also possess enhanced anti-inflammatory benefits. Therefore, the object of this study was to assess the capacity of HGSL DHLs to alleviate oxidative stress and inflammation in LPS-stimulated RAW264.7 cells, with pak choi serving as the parental control. 

## 2. Materials and Methods

### 2.1. Plant Materials and Sample Preparation 

The production of HGSL DHLs and their deposition in the NCBI database and the Korea Agricultural Culture Collection were previously outlined [20,21]. The plant materials employed in this study were sourced from the same samples harvested and prepared during March and April 2020, consistent with our earlier investigations [20,21]. Briefly, both pak choi and three HGSL DHLs (DH005, DH014, and DH026) were cultivated for 6 weeks in a greenhouse located at the National Institute of Agricultural Sciences (Jeonju, Republic of Korea). The growth conditions included a 16 h photoperiod at 24 °C [20]. Fresh leaves from the 6-week-old plants were rapidly lyophilized using an FDU-2110 freeze dryer (EYELA, Tokyo, Japan) [20,21]. Each freeze-dried sample (2 g) underwent extraction with 50 mL of deionized distilled water through 1 h of sonication, followed by filtering using Minisart^®^ 0.45 µm syringe filters (Sartorius, Göttingen, Germany) [20,21]. The effectiveness of this sample preparation was validated using GSL content determination through a high-performance liquid chromatography system with a 150 × 3.0 mm inner diameter and 3 µm particle size of an Inertsil ODS-3 column (GL Science, Tokyo, Japan) as described in prior publications [20,21]. The quantification of GSL contents involved the use of sinigrin as an external standard, following the established protocols [20,21]. Additionally, GLS hydrolysis products were quantified following natural hydrolysis with endogenous myrosinase [20]. The total GSL contents were determined as 44.12 ± 2.86, 56.06 ± 3.28, and 57.04 ± 1.54 for DH005, DH014, and DH026, respectively [20]. Correspondingly, the GSL hydrolysis products of DH005, DH014, and DH026 were measured at 709.8 ± 16.7, 870.3 ± 37.6, and 783.9 ± 23.5, respectively [20]. Notably, these values represented fold changes of 5.1, 6.3, and 5.6, respectively, when compared to pak choi, which exhibited GSL hydrolysis products of 139.2 ± 3.6 [20].

### 2.2. Cell Culture and Reagents

RAW264.7 mouse macrophages were grown as described in a previous study [5]. Lipopolysaccharides (LPSs) were procured from Sigma-Aldrich (L4391, St. Louis, MO, USA) [5].

### 2.3. Cell Viability Assays

The cytotoxicity of HGSL DHLs was evaluated using MTT (3-(4,5-dimethylthiazol-2-yl)- and 5-diphenyltetrazolium bromide) assays. The cells were seeded in 96-well plates with a density of 1 × 10^4^ cells/well and incubated overnight. Subsequently, the cells were treated with HGSL DHLs’ extracts. After 24 h of treatment, MTT assays were performed as previously described [5]. Briefly, a solution containing 20 μL of MTT (2 mg/mL) was introduced to each well and allowed for incubating for 4 h at 37 °C [5]. The absorbance at 570 nm was subsequently gauged using a microplate reader [21]. 

### 2.4. Nitric Oxide Assays

The level of nitric oxide (NO) secreted by RAW264.7 cells was determined using the same NO detection kit as described previously [5]. For cell seeding, RAW264.7 cells (3 × 10^5^ cells/well) were distributed into 6-well plates and allowed to incubate overnight [5]. Post pretreatment with either HGSL DHLs or *N*^G^-monomethyl L-arginine acetate salt (L-NMMA, a NO synthase (NOS) inhibitor) (Sigma-Aldrich) for 1 h, the cells were subsequently subjected to LPS stimulation for a duration of 24 h. The ensuing collection of culture supernatants facilitated the assessment of NO concentration. The determination of the NO concentration was thrice performed in triplicate, employing the procedures provided by the manufacturer and as previously outlined [5]. 

### 2.5. 2,2-Diphenyl-1-Picrylhydrazyl (DPPH) and 2,2′-Azino-bis (3-Ethylbenzothiazoline-Sulfonic Acid) (ABTS) Free Radical Scavenging Activity

The evaluation of free radical scavenging activity attributed to HGSL DHLs encompassed the utilization of the 2,2-diphenyl-1-picrylhydrazyl (DPPH) and 2,2′-Azino-bis (3-ethylbenzothiazoline-sulfonic acid) (ABTS) assays. For the DPPH assay, HGSL DHL samples were diluted to the required concentration and mixed with an equal volume of a 100 μM DPPH solution. After 15 min of reaction time under subdued lighting, their absorbances were taken at 515 nm, employing a microplate reader (Molecular Devices, CA, USA) [5]. We used ascorbic acid as a positive control. 

For the ABTS assay, 7 mM ABTS and 2.4 mM ammonium peroxodisulfate solutions were prepared. Both solutions were mixed at a 1:1 ratio and allowed to react overnight to generate the ABTS radical. The ABTS radical solution was then diluted to an absorbance of 0.7 ± 0.02 at 734 nm. The HGSL DHL samples were mixed with an equal volume of the ABTS radical solution and kept in darkness for 30 min at room temperature. The absorbance was determined at 734 nm. 

The free radical scavenging activity was calculated using the following formula: DPPH or ABTS radical scavenging activity (%) = (A_blank_ − A_sample_)/A_blank_ × 100, where A_blank_ represents the absorbance of the DPPH or ABTS solution without the test sample, and A_sample_ represents the absorbance of the test sample [5]. 

### 2.6. Measurement of Intracellular ROS

Intracellular ROS levels were determined with 2,7-dichlorofluorescein diacetate (DCF-DA) (Sigma-Aldrich, #D6883) as described earlier [5]. Cells were pre-incubated with HGSL GSLs or L-NMMA for 1 h, followed by stimulation with LPS (10 ng/mL) for 18 h. After the stimulation period, the cells were stained with 20 µM DCF-DA for 30 min at 37 °C in darkness [5]. The stained cells were then harvested and washed twice with PBS, and then 10,000 cells were analyzed using flow cytometry, following the procedure provided in a prior publication [5].

### 2.7. Real-Time Quantitative Reverse Transcription Polymerase Chain Reaction (qRT-PCR)

RAW264.7 cells (5 × 10^5^ cells/well) in 6-well plates were pretreated with HGSL DHLs for 1 h before treatment with LPS (10 ng/mL) for 8 h. Total RNA isolated from the treated cells were used for subsequent reverse transcription (RT) to cDNA, adhering to methodologies outlined in our previous research [21]. The amplification of each cDNA and its subsequent monitoring were carried out, in adherence to the protocol outlined previously [5]. The specific primers for *Hmox1*, *Gclc*, *Nqo1*, *Il6*, *Tnf*, *Il1b*, and *Gapdh* were previously described [5]. 

### 2.8. Preparation of Nuclear and Cytosolic Extracts

The cytoplasmic extraction buffer was prepared by combining 10 mM KCl, 0.2 mM EDTA, and 1.5 mM MgCl_2_, while the nuclear extraction buffer was prepared by combining 30 µL of ice-cold 20 mM HEPES (pH 7.9), 420 mM NaCl, 1.5 mM MgCl_2_, 20% (*v/v*) glycerol, and 0.2 mM EDTA. Additionally, 0.5 mM DTT and 0.2 mM phenylmethylsulfonyl fluoride (PMSF) were immediately added to the nuclear extraction buffer before use, as previously described [5]. 

RAW264.7 cells (1.5 × 10^6^ cells/plate) in 100 mm plates were pretreated with HGSL DHLs for 1 h before being stimulated with LPS (10 ng/mL) for 2 h. The cells were lysed with 150 µL of the cytosolic extraction buffer at 4 °C for 10 min, and the lysates were subjected to centrifugation at 14,000× *g* for 5 min. The resulting supernatants were collected and preserved as cytosolic extracts under −70 °C [5]. The pellet obtained from the centrifugation was resuspended in 50 µL of the ice-cold nuclear extraction buffer. Following a 30 min incubation at 4 °C, the lysates underwent centrifugation at 14,000× *g* for 10 min. Subsequently, the resulting supernatants were preserved as nuclear extracts at a temperature under −70 °C [22]. The concentrations of the extracts were gauged utilizing a BCA kit (Bio-Rad, Richmond, CA, USA) [5].

### 2.9. Immunoblotting Analysis

Cells underwent a 1 h pretreatment with HGSL DHLs, followed by an incubation with LPS (10 ng/mL) for 1~24 h. The preparation of nuclear, cytoplasmic, and whole-cell extracts was executed, with subsequent Western blotting performed in accordance with a prior description [21]. Primary antibodies targeting NRF2, Lamin B1, HMOX1, cyclooxygenase (COX) 2, inducible NOS (iNOS), STAT3, phospho (p)-STAT3, p65, p-p65, p38 MAPK (p38), p-p38, JUN N-terminal Kinase (JNK), p-JNK, and β-actin were previously referenced [5,21]. HRP-conjugated secondary antibodies anti-mouse and rabbit IgG were provided by Jackson Immuno Research Laboratories, Inc. (West Grove, PA, USA) [5,21]. ImageJ software (V1.53k, https://imagej.nih.gov/ij/, accessed on 5 November 2021) was utilized to analyze the density of protein bands. 

### 2.10. Statistical Analysis

Each experiment was independently conducted at least three times. The statistical analysis was performed using GraphPad Prism version 5.02 for Windows (GraphPad Software, San Diego, CA, USA). The data are presented as the mean ± the standard error of the mean (SEM), and the statistical analysis involved the application of a one-way analysis of variance (ANOVA), followed by Dunnett’s tests for the identification of significant differences among groups. A *p*-value of less than 0.05 was adopted as the threshold for statistical significance.

## 3. Results

### 3.1. HGSL DHL Extracts Reduce LPS-Induced Production of Pro-Inflammatory Mediators

**Figure 1:**
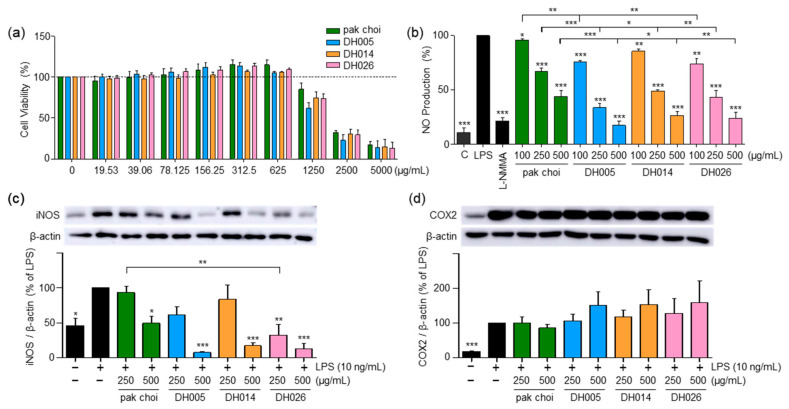
Effect of high-glucosinolate-containing doubled haploid lines (HGSL DHLs) (DH005, DH014, and DH026) on RAW264.7 cell viability and lipopolysaccharide (LPS)-induced pro-inflammatory mediator expression. (**a**) Cell viability was assessed by treating RAW264.7 cells with HGSL DHLs or the pak choi extract for 24 h at indicated concentrations, followed by MTT assays. (**b**) RAW264.7 cells were pretreated with HGSL DHLs or the pak choi extract for 1 h and stimulated with LPS for 24 h. NO production was measured in the culture media using the Griess assay. (**c**,**d**) Cells were pretreated with L-NMMA (200 μM), HGSL DHLs, or the pak choi extract for 1 h and stimulated with LPS for 24 h. The protein expression levels of iNOS and COX2 were assessed with Western blotting. The expression of iNOS and COX2 was quantified using ImageJ software V1.53k and normalized to the β-actin protein levels. Data are presented as the mean ± standard error of the mean (SEM) with statistical significance as * *p* < 0.05, ** *p* < 0.01, and *** *p* < 0.001 compared to the LPS-only or pak choi-treated group.

We initiated our investigation by assessing the cytotoxicity of HGSL DHLs in RAW264.7 cells using the MTT assay. The goal was to establish non-toxic concentrations of the extracts for subsequent experiments within the same cell line. As shown in Figure 1a, both pak choi and HGSL DHLs exhibited no cytotoxic effects within the concentration range of 19.53–625 μg/mL, as evidenced by the absence of a significant impact on cell viability. Consequently, concentrations lower than 625 μg/mL were chosen for further experiments in the cells.

LPS stimulation in RAW264.7 cells leads to the release of NO, a pro-inflammatory mediator and a reactive radical that plays a crucial role in the development and progression of inflammatory disorders [22]. As depicted in Figure 1b, HGSL DHLs exhibited a dose-dependent reduction in LPS-induced NO production, consistently outperforming the effects of pak choi (parental control). Notably, DH005 at 500 μg/mL demonstrated the most potent inhibitory effect, with an 82.35% reduction. Other HGSL DHLs also demonstrated substantial inhibitory effects, with DH014 and DH026 showing reductions of 73.26% and 76.12%, respectively, compared to pak choi’s 55.87% inhibition at the same concentration. Moreover, cells treated with 500 μg/mL of HGSL DHLs displayed NO levels comparable to those of 200 μM L-NMMA, the positive control, and even approached the levels of LPS-untreated negative control cells (Figure 1b).

Concomitant with the decline in NO levels, we examined the expression of iNOS, a protein implicated in NO production, and COX2, a protein that mediates inflammation through prostaglandin synthesis [4]. As shown in Figure 1c, HGSL DHLs dose-dependently reduced LPS-induced iNOS expression. All HGSL DHLs exhibited greater efficacy compared to pak choi, with DH026 yielding statistically significant inhibitory effects. This observation aligns with the diminished NO production, confirming that the reduction in iNOS expression correlated with decreased NO levels. However, no significant alteration was observed in COX2 expression (Figure 1d). Collectively, these results suggest that HGSL DHLs possess potential as potent antioxidant and anti-inflammatory agents.

### 3.2. HGSL DHLs Exhibit Antioxidant Activity

**Figure 2:**
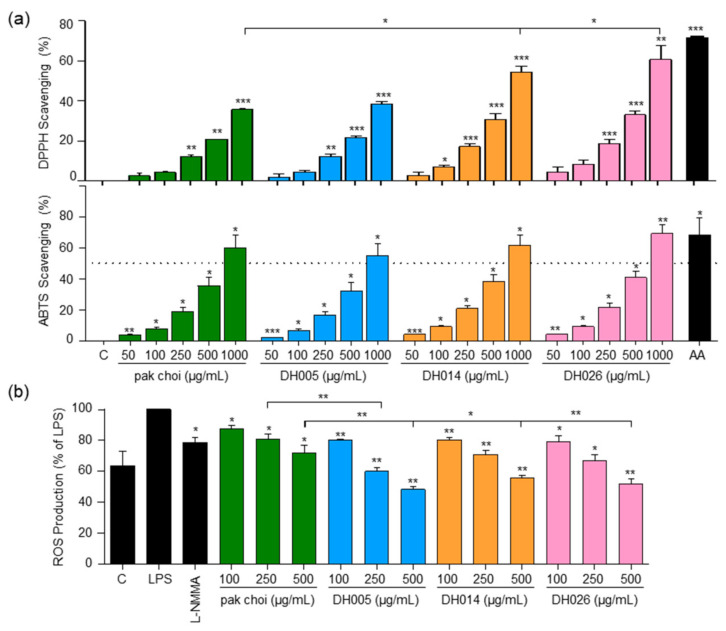
Antioxidant activity and intracellular ROS levels in response to HGSL DHLs (DH005, DH014, and DH026). (**a**) DPPH and ABTS radical scavenging activity of DH005, DH014, and DH026 compared to pak choi and ascorbic acid (AA, 100 µM) as a positive control. (**b**) RAW264.7 cells were preincubated with L-NMMA (200 μM), HGSL DHLs, or the pak choi extract for 1 h, followed by stimulating with LPS (10 ng/mL) for 16 h. Intracellular ROS production was determined with 2,7-dichlorofluorescein diacetate (DCF-DA) staining. Data are presented as the mean ± SEM with statistical significance indicated as * *p* < 0.05, ** *p* < 0.01, and *** *p* < 0.001 compared to the control, LPS-only, or pak choi-treated group.

To evaluate the antioxidant activity, we assessed the ability of HGSL DHLs to scavenge DPPH and ABTS free radicals using the respective assays. Both HGSL DHLs and pak choi demonstrated a dose-dependent scavenging effect on DPPH and ABTS radicals. Importantly, all HGSL DHLs exhibited superior radical scavenging activity compared to pak choi (Figure 2a). While statistical significance was achieved only in DPPH scavenging for DH014 and DH026, DH026 at 1000 μg/mL showed the most substantial DPPH radical scavenging activity at 59.49%, comparable to 100 μM ascorbic acid (71.64%) (Figure 2a). Moreover, DH026 displayed the highest ABTS radical scavenging activity, slightly outperforming ascorbic acid. Additionally, the ABTS scavenging activity of DH005 and DH014 at 1000 μg/mL approached that of 100 µM ascorbic acid (Figure 2a).

In addition to the in vitro antioxidant tests, we investigated the impact of HGSL DHLs on intracellular ROS levels in LPS-stimulated macrophages (Figure 2b). The proper regulation of ROS generation is crucial for modulating the inflammatory response [23]. While the positive control, L-NMMA, exhibited a 21.2% inhibition of ROS, pak choi at 500 μg/mL showed a 28.2% inhibition. Remarkably, HGSL DHLs at the same concentrations further reduced ROS levels, with DH005, DH014, and DH026 yielding reductions of 51.8%, 44.5%, and 48%, respectively. Notably, ROS levels in cells treated with 500 μg/mL HGSL DHLs were even lower than those in the negative control cells lacking LPS stimulation. These results indicated the robust antioxidant capacity of HGSL DHLs.

### 3.3. HGSL DHLs Enhance the Expression of Antioxidant Enzymes through NRF2 Activation

**Figure 3:**
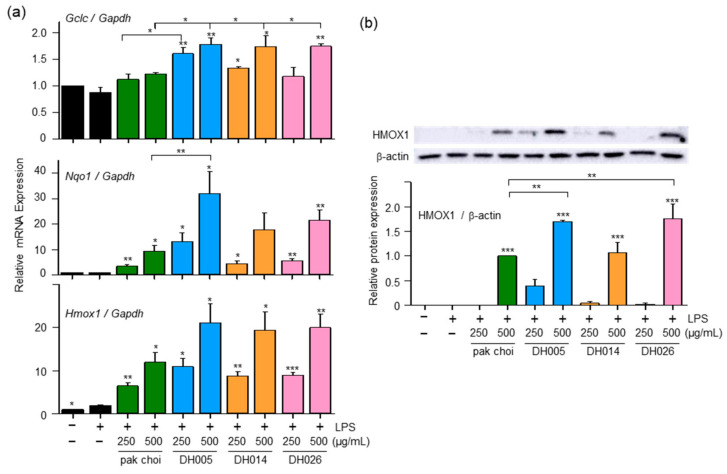
Effect of DH005, DH014, and DH026 on antioxidant gene expression. RAW264.7 cells were pretreated with HGSL DHLs or the pak choi extract for 1 h and stimulated with LPS (10 ng/mL) for 8 h. (**a**) The mRNA levels of *Gclc*, *Nqo1*, and *Hmox1* were measured with real-time qRT-PCR and normalized to *Gadph* mRNA levels. (**b**) HMOX1 protein expression was detected with Western blotting. The expression of HMOX-1 was quantified using ImageJ software and normalized to β-actin protein levels. Data are presented as the mean ± SEM from at least three independent experiments with statistical significance indicated as * *p* < 0.05, ** *p* < 0.01, and *** *p* < 0.001 compared to the LPS-only or pak choi-treated group.

To investigate the effects of HGSL DHLs on antioxidant enzymes, we assessed the expression of GCLC, NQO1, and HMOX1, which are involved in the antioxidant defense against ROS and oxidative stress [6]. As shown in Figure 3a, LPS alone had a minimal effect on the expression of these genes, whereas HGSL DHLs significantly upregulated the mRNA levels of *Gclc*, *Nqo1*, and *Hmox1* in a dose-dependent manner, exhibiting stronger effects compared to pak choi. Notably, *Nqo1* and *Hmox1* showed a robust response to HGSL DHL treatment. DH005 was particularly effective, with more than a 37-fold increase in *Nqo1* expression and an 11-fold increase in *Hmox1* expression compared to the LPS-only treatment group. These values were 3.4- and 1.76-fold higher than those observed with pak choi, respectively. Additionally, DH005 and DH026 exhibited elevated levels of HMOX1 protein compared to pak choi (Figure 3b), further confirming the gene expression results.

**Figure 4:**
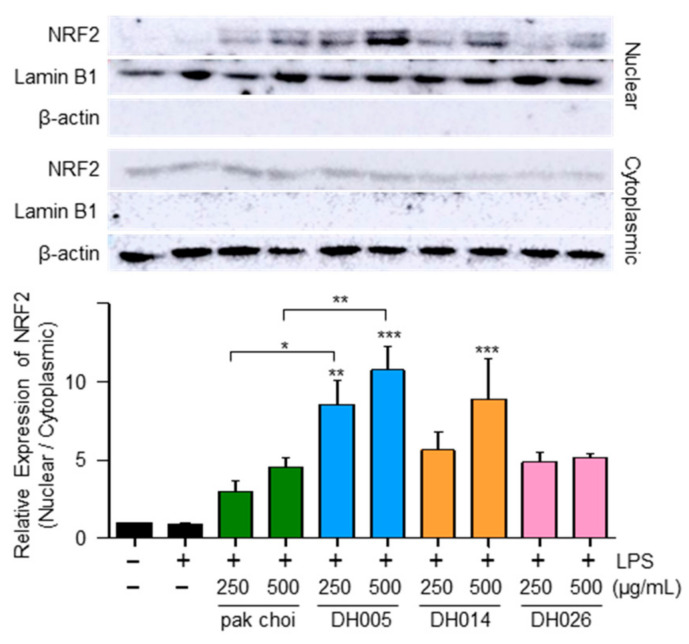
Effect of HGSL DHLs on NRF2 activation. RAW264.7 cells were pretreated with DH005, DH014, DH026, or the pak choi extract for 1 h and stimulated with LPS (10 ng/mL) for 2 h. The levels of NRF2 were measured using Western blotting in cytoplasmic and nuclear lysates. The nuclear translocation of NRF2 was quantified by comparing the levels of NRF2 in the nucleus and cytoplasm. Data are presented as the mean ± SEM from at least three independent experiments with statistical significance indicated as * *p* < 0.05, ** *p* < 0.01, and *** *p* < 0.001 compared to the LPS-only or pak choi-treated group.

Considering that NRF2 as a transcription factor plays a critical role in regulating the expression of antioxidant enzymes [10], we examined the nuclear localization of NRF2 as a potential mechanism underlying the antioxidant effects of HGSL DHLs. As shown in Figure 4, both HGSL DHLs and pak choi promoted the nuclear translocation of NRF2. Notably, DH005 showed the most significant enhancement in NRF2 nuclear translocation, while DH014 and DH026 outperformed pak choi. These observations were consistent with the effects of HGSL DHLs on the expression of antioxidant enzymes. Together, these results suggest that HGSL DHLs increase antioxidant enzyme expression through NRF2-mediated gene induction.

### 3.4. HGSL DHLs Induce Phosphorylation of p38

**Figure 5:**
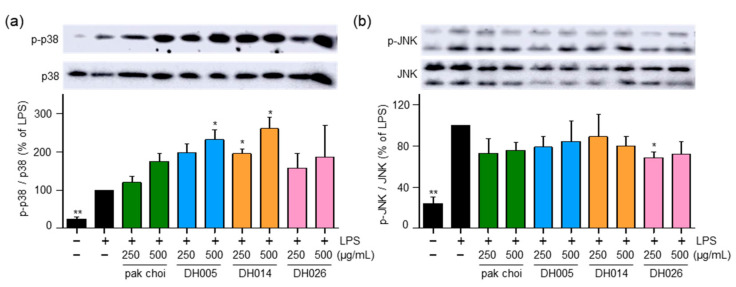
Effect of HGSL DHLs on MAPK signaling pathways. RAW264.7 cells were pretreated with DH005, DH014, DH026, or the pak choi extract for 1 h and stimulated with LPS (10 ng/mL) for 1 h. Phosphorylation levels of JNK1/2 and p38 MAPK were detected using Western blotting and normalized to total JNK and p38 MAPK, respectively. Data are presented as the mean ± SEM with statistical significance indicated as * *p* < 0.05 compared to the LPS-only treated group.

The MAPK pathway is known to play a role in the activation of NRF2 and the promotion of its phosphorylation and nuclear translocation, leading to the induction of downstream antioxidant enzymes [6]. Therefore, we investigated the activity of p38 and JNK. Interestingly, DH005 and DH014 treatment increased the phosphorylation of p38, while DH026 and pak choi showed minimal effects on p38 activation (Figure 5a). Conversely, all HGSL DHLs and pak choi extracts had no significant impact on the activation of JNK (Figure 5b). These findings suggest that HGSL DHLs may induce the antioxidant enzyme system through the activation of the p38-NRF2 signaling pathway.

### 3.5. HGSL DHLs Reduce LPS-Induced Pro-Inflammatory Cytokines and Inhibit LPS-Mediated STAT3 Activation

**Figure 6:**
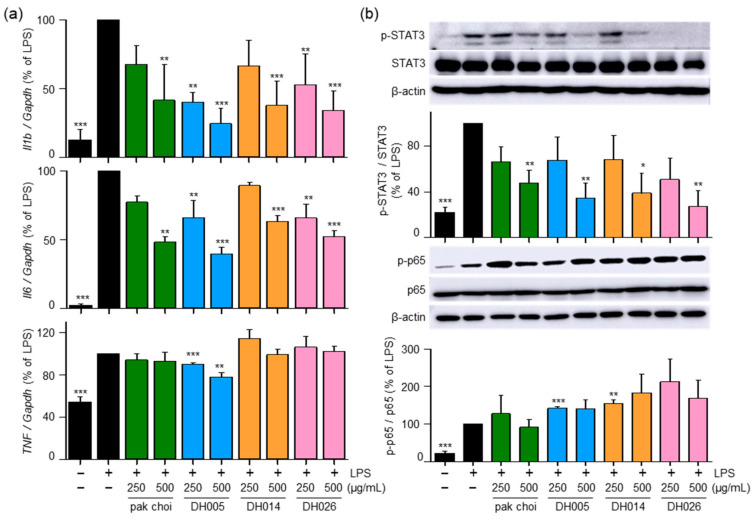
Effects of HGSL DHLs on pro-inflammatory cytokine gene expression and the phosphorylation of STAT3 and NF-κB. (**a**) RAW264.7 cells were pretreated with DH005, DH014, DH026, or the pak choi extract for 1 h and stimulated with LPS (10 ng/mL) for 8 h. The mRNA expression levels of *Il1b*, *Il6*, and *Tnf* were measured using real-time qRT-PCR and normalized to *Gapdh* mRNA levels. (**b**) RAW264.7 cells were pretreated with HGSL DHLs or pak choi for 1 h and stimulated with LPS (10 ng/mL) for 5 or 24 h. Phosphorylation levels of NF-κB p65 and STAT3 were detected with Western blotting and normalized to total p65 and STAT3, respectively. Data are presented as the mean ± SEM from three independent experiments. * *p* < 0.05, ** *p* < 0.01, and *** *p* < 0.001 denote statistically significant differences compared to the LPS-only treated group.

To further investigate the anti-inflammatory effects of HGSL DHLs and their underlying mechanisms, we examined mRNA expression levels of pro-inflammatory genes encoding IL-1β, IL-6, and TNF-α. As shown in Figure 6a, both HGSL DHLs and pak choi effectively reduced the LPS-induced mRNA expression of *Il6* and *Il1b*, with DH005 exhibiting the strongest inhibitory effect. However, only DH005 treatment significantly decreased the LPS-induced mRNA expression of *Tnf*, while the others showed no significant effect. 

Furthermore, we investigated the effects of HGSL DHLs on the LPS-induced activation of NF-κB and STAT3, which are transcription factors involved in regulating the expression of genes encoding pro-inflammatory cytokines as well as COX2 and iNOS [4]. Interestingly, HGSL DHLs inhibited the LPS-mediated phosphorylation of STAT3, while no effects were observed on the LPS-mediated p65 phosphorylation (Figure 6b). These results suggest that the anti-inflammatory effects of HGSL DHLs are mediated by the reduction in pro-inflammatory cytokines and the inhibition of STAT3 activation.

## 4. Discussion

Vegetables from the *Brassica rapa* species, including pak choi, *Brassica rapa* var. *glabra* (bomdong), *Brassica rapa* subsp. *pekinensis* (napa cabbage), *Brassica rapa* subsp. *rapa* (turnip), and yellow sarson, have long been recognized for their health benefits due to their rich nutritional profiles and bioactive compounds [13]. These vegetables contain essential nutrients such as vitamins, minerals, carotenoids, flavonoids, and dietary fiber, which contribute to their antioxidant and anti-inflammatory properties [13,24]. Notably, *Brassica rapa* vegetables are unique as they are rich in GSLs, sulfur-containing compounds responsible for their distinctive flavors and potential health benefits [25]. In this study, we focused on HGSL DHLs developed as leafy vegetables to eat leaves from the intercrossing of *Brassica rapa* subspecies, yellow sarson and pak choi [20]. We aimed to investigate the antioxidant and anti-inflammatory effects of HGSL DHLs compared to their parental control, pak choi, and our results demonstrated the remarkable potential of HGSL DHLs in these aspects.

Our findings revealed that HGSL DHLs effectively reduced the production of LPS-induced NO, surpassing the inhibitory effects observed with pak choi. This reduction in NO levels was associated with the downregulation of iNOS expression, confirming the anti-inflammatory effects of HGSL DHLs (Figure 1). Furthermore, HGSL DHLs demonstrated the ability to modulate the expression of pro-inflammatory cytokines (Figure 6a). The mRNA levels of *Il1b* and *Il6* were significantly decreased by HGSL DHLs, with DH005 exhibiting the most potent inhibitory effect. Interestingly, HGSL DHLs also inhibited the LPS-induced phosphorylation of STAT3 (Figure 6b), a transcription factor involved in the regulation of pro-inflammatory cytokines. This inhibition correlated with the decreased expression of *Il1b* and *Il6*, suggesting that the anti-inflammatory effects of HGSL DHLs may be mediated, at least in part, by the inhibition of STAT3 activation. However, only DH005 treatment showed a slight reduction in the mRNA expression of *Tnf*. Moreover, no significant effect on NF-κB was observed (Figure 6). NF-κB is a crucial modulator of the inflammatory response, and its inhibition is often related to the downregulation of pro-inflammatory factors [26]. While HGSL DHLs did not affect NF-κB phosphorylation, it is worth noting that SFN, a metabolite of GSLs that present in HGSL DHLs, has been reported to target NF-κB activity by interfering with its DNA binding without affecting its expression and nuclear translocation [27]. Thus, the reduction in pro-inflammatory cytokines using HGSL DHLs without affecting NF-κB may be attributed to the presence of SFN. Collectively, our findings suggest that HGSL DHLs have the potential to exert anti-inflammatory effects by attenuating the production of pro-inflammatory cytokines.

In addition to their anti-inflammatory properties, HGSL DHLs exhibited remarkable antioxidant activity (Figure 2). Our results demonstrated the dose-dependent DPPH and ABTS radical scavenging capacity of HGSL DHLs, surpassing that of pak choi. Notably, DH026 exhibited the highest radical scavenging activity at the concentration of 1000 μg/mL, comparable to that of ascorbic acid (100 μM), a well-known antioxidant. These results highlight the potential of HGSL DHLs as effective antioxidants. Furthermore, HGSL DHLs promoted the nuclear translocation of NRF2 (Figure 4), a transcription factor crucial for the induction of antioxidant enzymes. The upregulation of *Gclc*, *Nqo1*, and *Hmox1* mRNA levels, as well as the elevated protein levels of HMOX1, supported the activation of the NRF2 pathway by HGSL DHLs (Figure 3). These findings suggest that HGSL DHLs enhance the antioxidant defense system by activating NRF2-mediated gene induction. Moreover, HGSL DHLs activated the p38 MAPK signaling pathway (Figure 5), which has been implicated in the regulation of NRF2 phosphorylation and nuclear translocation [6,28]. The phosphorylation of p38 was significantly increased with DH005 and DH014, suggesting their involvement in activating NRF2 and subsequent antioxidant enzyme induction. In contrast, JNK phosphorylation remained unaffected by HGSL DHL treatment. These findings suggest that HGSL DHLs activate the p38-NRF2 signaling pathway to enhance antioxidant enzyme expression. However, our observation regarding the significant elevation of NRF2 nuclear translocation with certain concentrations of both pak choi and HGSL DHLs, despite only an increasing trend of p38 phosphorylation, suggests the possible contribution of other molecular mechanisms to dissociate NRF2 from KEAP1 [10]. It is worth noting that NRF2 has been reported to inhibit the inflammatory response by suppressing the expression of pro-inflammatory cytokines [29]. SFN, a metabolite of GSLs that is highly abundant in HGSL DHLs, has been reported to reduce inflammation via an NRF2-dependent mechanism [19]. Therefore, the enhanced antioxidant properties may be closely related to their anti-inflammatory effects.

## 5. Conclusions 

In conclusion, our study provides valuable insights into the anti-inflammatory and antioxidant properties of HGSL DHLs derived from *Brassica rapa* subspecies, pak choi, and yellow sarson. The results suggest that HGSL DHLs possess the potential to modulate key molecular pathways involved in inflammation and oxidative stress. These findings suggest HGSL DHLs as health-promoting leafy vegetables to protect us against inflammation and oxidative-stress-related diseases. In addition, these results suggest them as promising candidates for the development of therapeutic interventions targeting inflammatory and oxidative diseases such as cancers and cardiovascular diseases. Further investigations are warranted to fully elucidate the precise mechanisms underlying the effects of HGSL DHLs and their potential applications in preclinical and clinical settings.

## Data Availability

The data presented in this study are available in the article.
